# Interstitial lung abnormalities after hospitalization for COVID‐19 in patients with cancer: A prospective cohort study

**DOI:** 10.1002/cam4.6396

**Published:** 2023-08-18

**Authors:** Sungryong Noh, Christopher Bertini, Isabel Mira‐Avendano, Maryam Kaous, Bela Patel, Saadia A. Faiz, Vickie R. Shannon, Diwakar D. Balachandran, Lara Bashoura, Roberto Adachi, Scott E. Evans, Burton Dickey, Carol Wu, Girish S. Shroff, Joanna‐Grace Manzano, Bruno Granwehr, Shannon Holloway, Kodwo Dickson, Alyssa Mohammed, Mayoora Muthu, Hui Song, D3CODE Team, Ashley Aroe, Thomas A. Aloia, II Lee Andrews, Kiran K. Badami, Janna A. Baganz, Pratibha Bajwa, Lora R. Baker, Gregory R. Barbosa, Hannah C. Beird, Matt Bourgeois, Kristy Brock, Elizabeth M. Burton, Juan Cata, Caroline Chung, Michael Cutherell, John Cuenca, Pierre B. Cyr, Bouthaina Dabaja, Hiba Dagher, Kevin M Daniels, Mary Domask, Giulio Draetta, Paul Edelkamp, Sarah Fisher, Katy Elizabeth French, Andrew Futreal, Maria Gaeta, Christopher Gibbons, Myrna Godoy, Drew Goldstein, Jillian Gunther, Cristhiam Hernandez, Kate Hutcheson, David Jaffray, Jeff Jin, Teny Matthew John, Trey Kell, Mark Knafl, Anai Kothari, Rayson C. Kwan, J. Jack Lee, Yue Liao, Jennifer Litton, Alex Liu, Kevin W. McEnery, Mary McGuire, Benjamin Mescher, Tejo Musunuru, Mayoora Muthu, Joseph Nates, Craig S. Owen, Priyadharshini Padmakumar, Melody Page, Nicholas Palaskas, Jay J. Patel, Sabitha Prabhakaran, Lucas Ramsey, Vinod Ravi, Ludivine Russell, Bilja Sajith, Paul A. Scheet, Stephanie Schmidt, Kenna R. Shaw, Sanjay Shete, Daniel P. Shoenthal, Lessley J. Stoltenberg, Ishwaria Subbiah, Chuck Suitor, Hussein Tawbi, Phillip Thompson, Anastasia Turin, Samir Unni, Benju Vicknamparampil, Max C. Weber, John Weinstein, Zoe Williams, Scott Eric Woodman, Mark C. Wozny, Carol Wu, Jia Wu, James C. Yao, Chingyi Young, Emily Yu, Steven Zatorski, Caroline Chung, Jia Wu, Lyndon Lee, Ying Jiang, Fareed Khawaja, Ajay Sheshadri

**Affiliations:** ^1^ Division of Critical Care, Pulmonary and Sleep Medicine McGovern Medical School Houston Texas USA; ^2^ Department of Internal Medicine McGovern Medical School at UT Health Houston Texas USA; ^3^ Department of Pulmonary Medicine The University of Texas MD Anderson Cancer Center Houston Texas USA; ^4^ Department of Thoracic Imaging The University of Texas MD Anderson Cancer Center Houston Texas USA; ^5^ Department of Hospital Medicine The University of Texas MD Anderson Cancer Center Houston Texas USA; ^6^ Department of Infectious Diseases, Infection Control, and Employee Health The University of Texas MD Anderson Cancer Center Houston Texas USA; ^7^ Data‐Driven Determinants for COVID‐19 Oncology Discovery Effort (D3CODE) Team The University of Texas MD Anderson Cancer Center Houston Texas USA; ^8^ Department of Radiation Oncology The University of Texas MD Anderson Cancer Center Houston Texas USA; ^9^ Department of Imaging Physics, Infection Control, and Employee Health The University of Texas MD Anderson Cancer Center Houston Texas USA

**Keywords:** COVID‐19, fibrosis, interstitial lung disease, pneumonia, post‐infectious pulmonary complication

## Abstract

**Introduction:**

Survivors of SARS‐CoV‐2 pneumonia often develop persistent respiratory symptom and interstitial lung abnormalities (ILAs) after infection. Risk factors for ILA development and duration of ILA persistence after SARS‐CoV‐2 infection are not well described in immunocompromised hosts, such as cancer patients.

**Methods:**

We conducted a prospective cohort study of 95 patients at a major cancer center and 45 patients at a tertiary referral center. We collected clinical and radiographic data during the index hospitalization for COVID‐19 pneumonia and measured pneumonia severity using a semi‐quantitative radiographic score, the Radiologic Severity Index (RSI). Patients were evaluated in post‐COVID‐19 clinics at 3 and 6 months after discharge and underwent comprehensive pulmonary evaluations (symptom assessment, chest computed tomography, pulmonary function tests, 6‐min walk test). The association of clinical and radiological factors with ILAs at 3 and 6 months post‐discharge was measured using univariable and multivariable logistic regression.

**Results:**

Sixty‐six (70%) patients of cancer cohort had ILAs at 3 months, of whom 39 had persistent respiratory symptoms. Twenty‐four (26%) patients had persistent ILA at 6 months after hospital discharge. In adjusted models, higher peak RSI at admission was associated with ILAs at 3 (OR 1.5 per 5‐point increase, 95% CI 1.1–1.9) and 6 months (OR 1.3 per 5‐point increase, 95% CI 1.1–1.6) post‐discharge. Fibrotic ILAs (reticulation, traction bronchiectasis, and architectural distortion) were more common at 6 months post‐discharge.

**Conclusions:**

Post‐COVID‐19 ILAs are common in cancer patients 3 months after hospital discharge, and peak RSI and older age are strong predictors of persistent ILAs.

## 1 INTRODUCTION

After SARS‐CoV‐2 infection, interstitial lung abnormalities (ILA),^1^ are often observed in survivors and may represent a pulmonary form of the post‐acute sequelae of COVID‐19 (PASC).^2^ Post‐COVID‐19 ILAs may be associated with long‐term pulmonary impairment and fibrosis, as was seen during the severe acute respiratory syndrome (SARS) coronavirus outbreak in 2003.^3^ Although, several studies have noted a high rate of post‐infection ILAs in survivors of SARS‐CoV‐2 hospitalization,^1^, ^4^ a clearer understanding of the factors that lead to post‐COVID‐19 ILAs is lacking. Severity of illness, age, and elevated serum inflammatory markers may increase the risk for the development of ILAs following COVID‐19 infection, but the findings vary depending upon the timing of ILA characterization and the composition of the post‐COVID‐19 cohort.^5^, ^6^

Cancer patients, particularly those with hematologic malignancies, are at high risk for death after developing SARS‐CoV‐2 pneumonia.^7^, ^8^ However, it is unclear whether the higher risk also contributes to an elevated incidence of post‐COVID‐19 ILAs than the general population, or if the elevated upfront mortality might result in a lower rate of long‐term ILAs due to attrition in the most severe cases. Furthermore, it is uncertain whether risk factors for post‐COVID‐19 ILAs are similar in cancer and non‐cancer patients. The purpose of this study was to measure the incidence of post‐COVID‐19 ILAs in cancer patients who survived to hospital discharge after SARS‐CoV‐2 pneumonia and determine risk factors for persistent ILAs.

## 2 METHODS

### 2.1 Study design and population

We conducted an analysis of a prospective cohort at a major cancer center (The University of Texas MD Anderson Cancer Center, MDACC; cancer cohort) and in patients without cancer at a tertiary referral center (The University of Texas Health Science Center at Houston, UTH; non‐cancer cohort). At MDACC, we enrolled consecutive patients of at least 18 years of age who had confirmed SARS‐CoV‐2 pneumonia confirmed by nucleic acid amplification testing from the nasopharynx or bronchoalveolar lavage and were hospitalized for SARS‐CoV‐2 pneumonia between 2020 and 2021 and survived to hospital discharge. Upon discharge, patients were systematically referred to a post‐COVID‐19 clinic and saw experts from pulmonary medicine or infectious diseases between June 2020 and June 2021, which corresponded to a full year following the initial wave of COVID‐19 in the United States. A comprehensive pulmonary evaluation occurred approximately 3 months after hospital discharge and included imaging and pulmonary testing.

**FIGURE 1:**
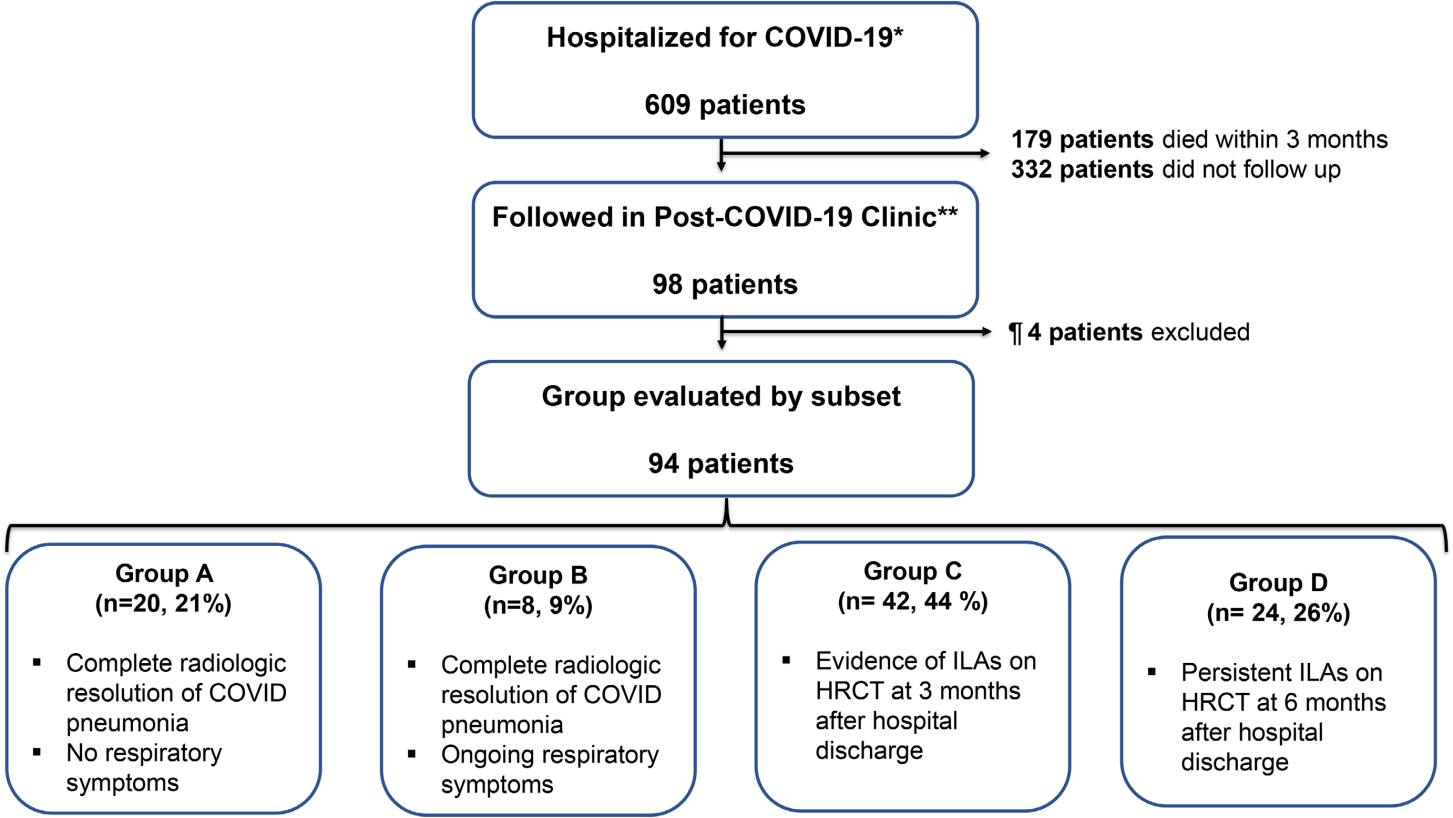
Selection of the final study cohort (*n* = 94).

**FIGURE 2:**
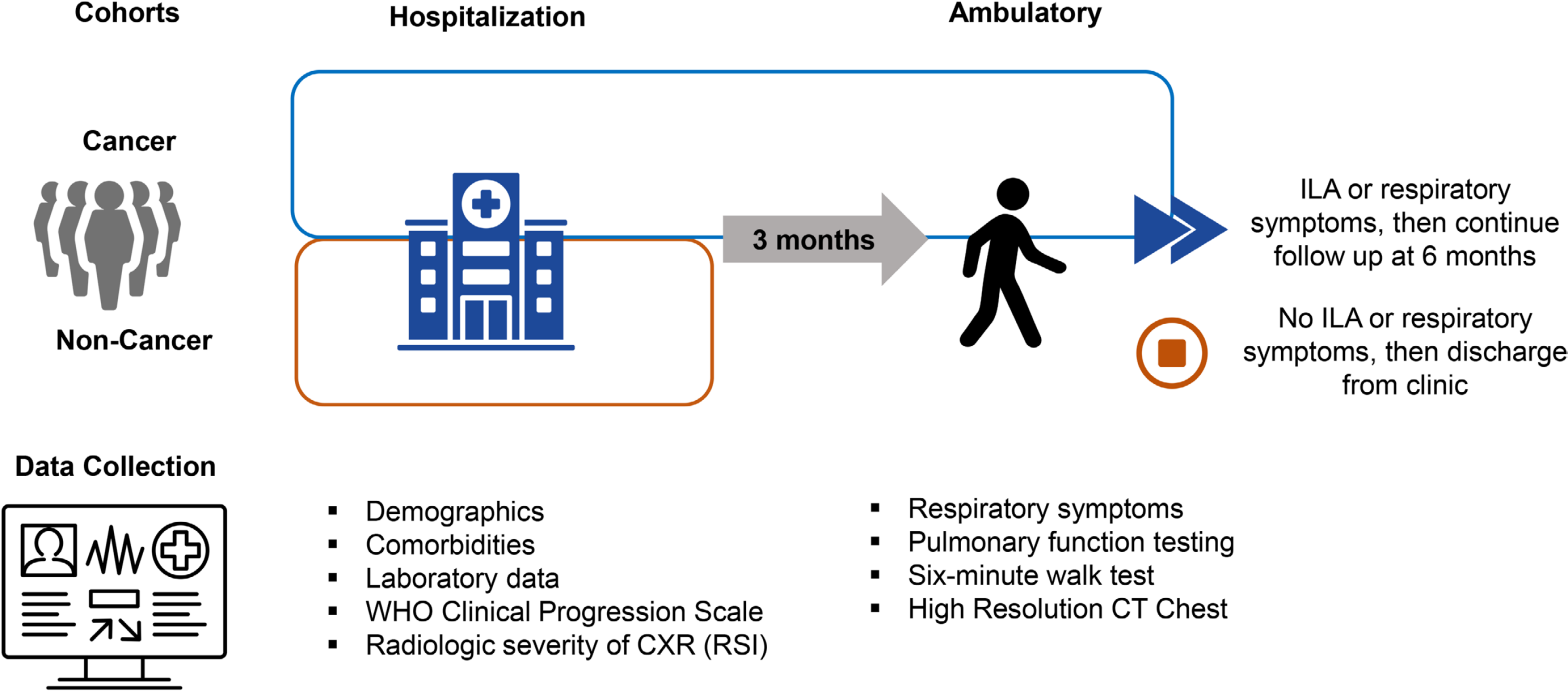
Schema for post‐COVID‐19 evaluations.

At the Center of Excellence for COVID‐19 care at UTH (COE‐UTH), patients with evidence of prior infection were evaluated regardless of need for hospitalization; symptomatic patients underwent pulmonary evaluations at the initial post‐COVID‐19 visit, followed by 3 and 6 months afterward. For this study, we only included patients who had been hospitalized at UTH for SARS‐CoV‐2 pneumonia in order to analyze all relevant data upon initial hospital admission. Figure 1 shows the selection of our study cohorts, and Figure 2 shows a diagram of post‐COVID‐19 evaluations. This project was approved by the institutional review boards (MDACC 2020‐0348, UTH HSC‐MS‐20‐0563).

### 2.2 Data collection

#### 2.2.1 Initial admission

**FIGURE 3:**
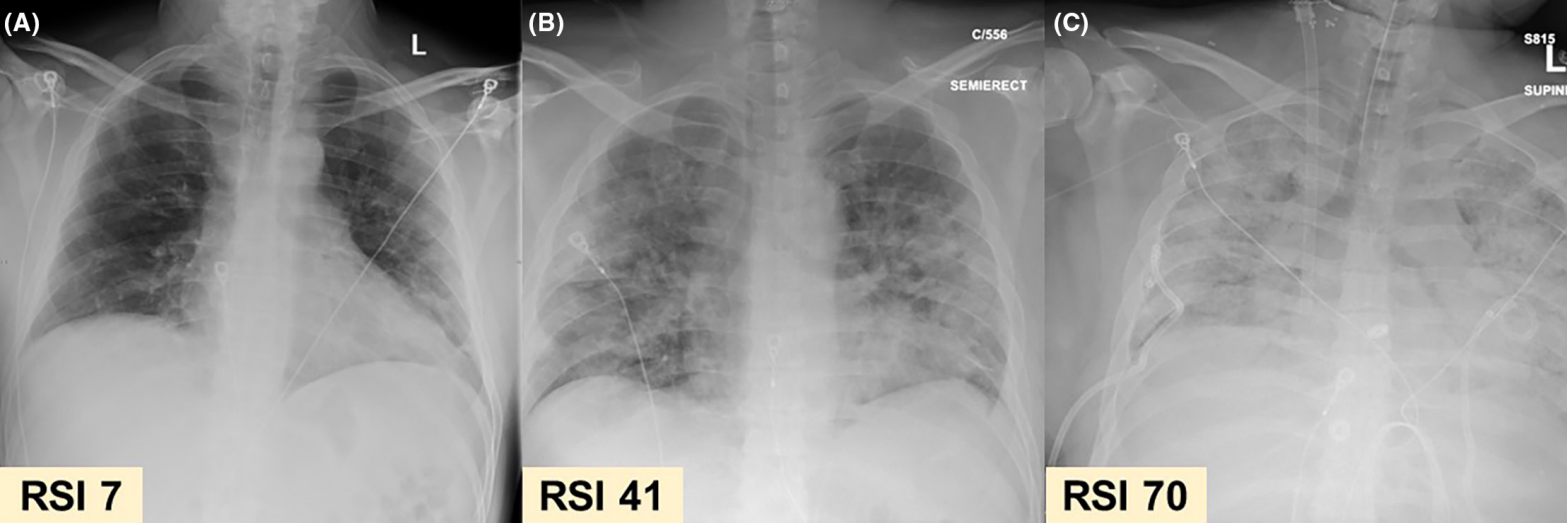
Representative images of a patient with mild (A, RSI 7), severe (B, RSI 41), and very severe (C, RSI 70) SARS‐CoV‐2 pneumonia.

Clinical data were extracted from the electronic health record and included: demographics; comorbidities; admission laboratory tests, focusing on complete blood counts with differentials, coagulation studies, and peak values for the acute phase reactants lactate dehydrogenase (LDH), C‐reactive protein (CRP), ferritin, d‐dimer, fibrinogen, and erythrocyte sedimentation rate (ESR); maximal oxygen requirement (need for non‐invasive or invasive positive pressure ventilation, the need for extracorporeal membrane oxygenation, ECMO); need for renal replacement therapy (RRT) during admission; mortality (in‐hospital, or once discharged, before any outpatient follow‐up). We calculated Charlson Comorbidity Index (CCI) using comorbidity data.^9^ Radiologic severity of the initial chest radiograph and the most severe chest radiograph within 28 days of admission was measured using the Radiologic Severity Index (RSI), a semi‐quantitative scoring tool that reproducibly measures the radiologic severity of pneumonia and is associated with mortality and other outcomes (Table 1).^10^, ^11^, ^12^ Each radiograph was scored by two readers (CB, SN), and the mean value was used for analyses. Figure 3 shows an example of COVID‐19 patients with varying degrees of peak radiological severity (RSI).

**TABLE 1:** Scoring algorithm for the radiologic severity index^a^

Predominant radiologic pattern in lung zone	Pattern score	Extent of volumetric radiologic involvement	Volumetric score
Normal lung	1	0%	0
Ground glass opacities	2	1%–24%	1
Consolidation	3	25%–49%	2
		50%–74%	3
		75%–100%	4

^a^ Radiologic severity index (RSI) scores are calculated by multiplying the predominant pattern for each lung zone by the extent of volumetric radiologic involvement for that zone. The sum of scores from all six zones gives the final RSI, ranging from 0 to 72.

The World Health Organization (WHO) clinical progression scale (Table 2) was used to measure the clinical severity of the initial SARS‐CoV‐2 pneumonia. Because all patients in this study were hospitalized and survived to hospital discharge, the values ranged from 4 (hospitalized; no oxygen therapy) to 9 (mechanical ventilation pO_2_/FiO_2_ < 150 and vasopressors, dialysis, or ECMO) for patients who survived to post‐COVID‐19 evaluation.^13^

**TABLE 2:** World Health Organization (WHO) clinical progression scale.

Patient state	Descriptor	Score
Uninfected	Uninfected; no viral RNA detected	0
Ambulatory mild disease	Asymptomatic; viral RNA detected	1
	Symptomatic; independent	2
	Symptomatic; assistance needed	3
Hospitalized: moderate disease	Hospitalized, no oxygen therapy	4
	Hospitalized; oxygen by mask or nasal cannula	5
Hospitalized: severe disease	Hospitalized; oxygen by NIV or high flow	6
	Intubation and mechanical ventilator, pO_2_/FiO_2_ ≥ 150 or SpO_2_/FiO_2_ ≥ 200	7
	Mechanical ventilation pO_2_/FiO_2_ < 150 (SpO_2_/FiO_2_ < 200) or vasopressors	8
	Mechanical ventilation pO_2_/FiO_2_ < 150 and vasopressors, dialysis, or ECMO	9
Dead	Dead	10

Abbreviations: ECMO, extracorporeal membrane oxygenation; FiO_2,_ fraction of inspired oxygen; NIV, non‐invasive ventilation; PO_2,_ partial pressure of oxygen; SpO_2_, oxygen saturation.

#### 2.2.2 Post‐COVID‐19 clinical evaluations

All cancer patients received a comprehensive pulmonary evaluation at the time of the initial post‐COVID‐19 follow‐up. Respiratory symptoms were captured at each visit including cough, shortness of breath, and chest pain/tightness. Low‐dose high‐resolution CT imaging (HRCT) scans were performed at all visits and reviewed by a thoracic radiologist. At MDACC, if no diagnostic imaging was being performed as part of their cancer care, patients underwent inspiratory, and expiratory HRCT. Specifically, pulmonary evaluation included: measurements of spirometry, including forced expiratory volume in 1 s (FEV_1_), forced vital capacity (FVC); plethysmography with total lung capacity (TLC); diffusing capacity for carbon monoxide (DLCO); 6‐min walk tests (6MWT) and measured distance (in meters) and the SpO2 nadir (%). Patients with no evidence of pulmonary impairment or ILAs on HRCT were discharged from the clinic, while those with symptoms or ILAs were seen at 6 and 12 months after hospital discharge.

**FIGURE 4:**
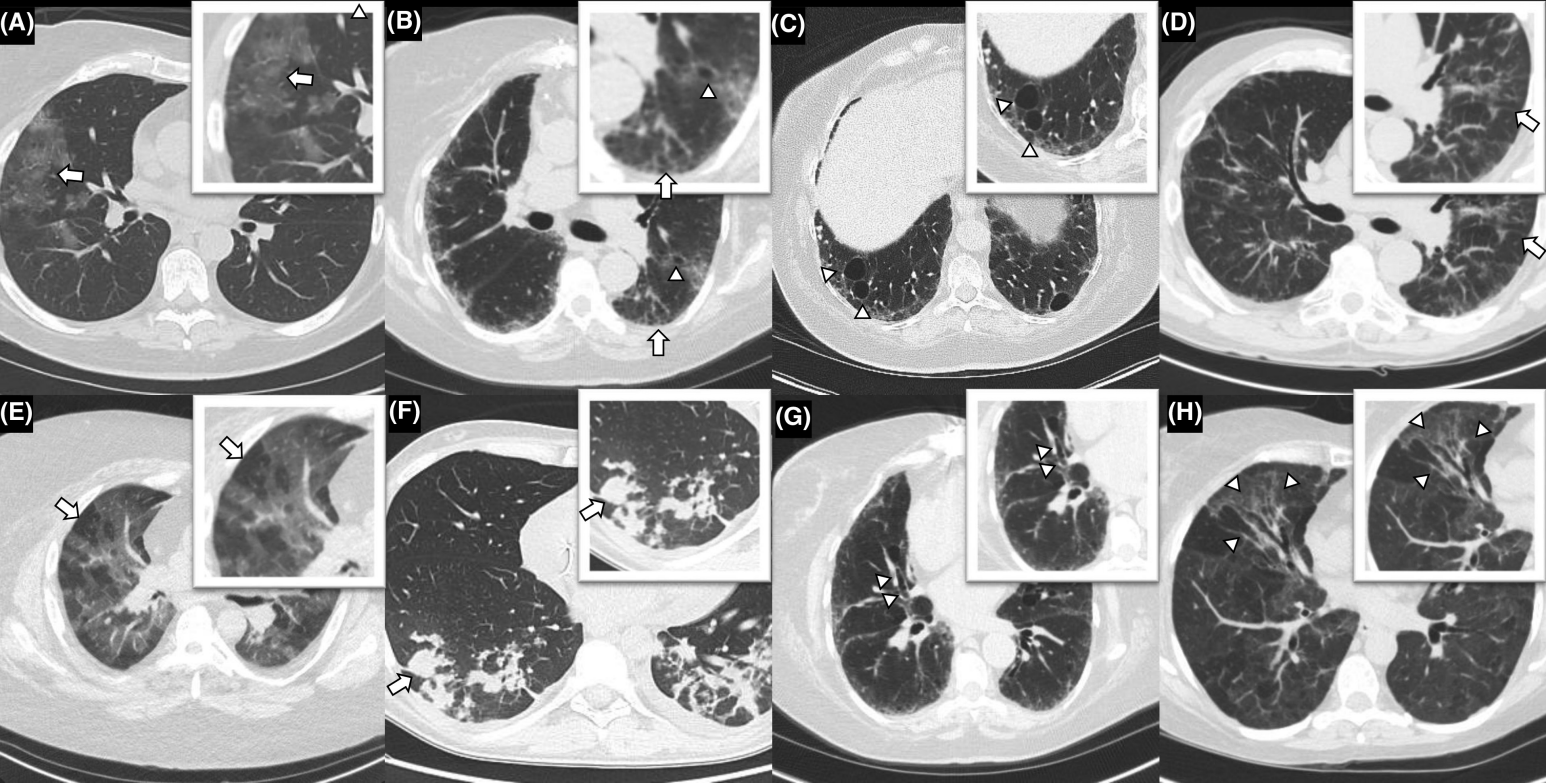
Representative images of interstitial lung abnormalities (ILAs). Arrows illustrate representative abnormalities, while the inset figure shows the abnormalities with 50% magnification. (A–C) ILAs seen at 3 months. (A) Ground‐glass opacities (GGOs). (B) Reticular opacities. (C) Reticular opacities and non‐emphysematous cysts. (D–H) Examples of ILAs seen at 6 months. (D) Reticular opacities and bronchiectasis. (E) Air trapping. (F) Lower lobe consolidation. (G) Honeycombing and traction bronchiectasis. (H) Architectural distortion and GGOs.

**FIGURE 5:**
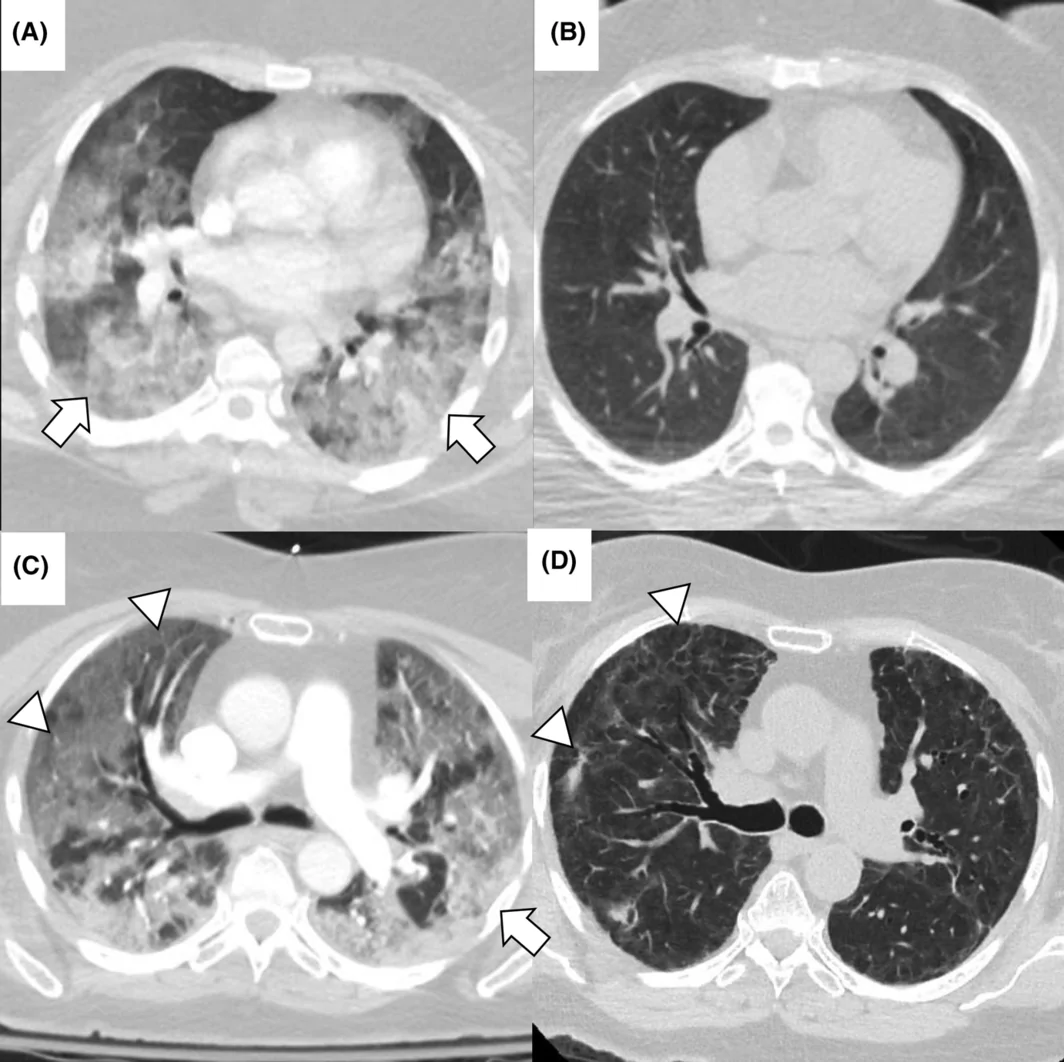
Representative images of two separate patients with transient (A, B) and persistent (C, D) ILAs. (A) Imaging from the initial SARS‐CoV‐2 pneumonia with complete resolution (B) 6 months after hospital discharge. (C) Imaging from the initial SARS‐CoV‐2 pneumonia in a patient with persistent ILAs (D) 6 months after hospital discharge, including reticular and ground‐glass opacities and architectural distortion, highlighted by the white arrows.

We measured the presence of ILAs at 3 and 6 months after hospital discharge. The adjudication of ILAs was reviewed by expert thoracic radiologists and an interstitial lung disease specialist (IM) reviewed chest HRCT and characterized the features of ILAs if present. Consistent with Fleischner Society definitions, we defined ILAs as the incidental finding of non‐dependent abnormalities that affected more than 5% of the cross‐sectional area of at least one of three lung zones on CT images.^14^, ^15^ ILAs were characterized as fibrotic when CT findings of architectural distortion, reticulation, traction bronchiectasis, and/or honeycombing are present (Figure 4). Ground glass opacities (GGO), tree‐in‐bud nodules, and mosaic attenuation were considered to be non‐fibrotic ILAs. We categorized patients into four groups according to ILAs and symptom status: Group A, complete resolution of radiologic and clinical respiratory symptom; Group B, no evidence of post‐COVID‐19 ILAs but had ongoing respiratory symptoms; Group C, evidence of ILAs at 3 months after hospital discharge regardless of symptoms, but resolution of radiologic changes by 6 months; Group D, presence of ILAs at 6 months regardless of symptoms after hospital discharge. In the event that a patient had evidence of ILAs prior to SARS‐CoV‐2 pneumonia, we only considered them to have ILAs at 3 or 6 months post‐discharge if the ILAs were newly evident after SARS‐CoV‐2 pneumonia. Figure 5 shows representative images from patients with transient (group C) and persistent (group D) ILAs.

### 2.3 Statistical analysis

Median and interquartile ranges (IQR) for continuous variables and analyzed continuous data using Wilcoxon rank‐sum tests were reported. Counts (*n*) and percentages (%) were used to describe categorical variables, and chi‐squared or Fisher's exact tests were used to analyze categorical data. The association of variables with post‐COVID‐19 ILAs at 3 and 6 months were measured using univariable logistic regression, and all variables with *p* ≤ 0.25 in univariable analyses were included into an initial multivariable model, and then used backward elimination to keep only variables with *p* ≤ 0.05 in the final model. The intraclass correlation coefficient was computed to assess the absolute agreement between two physicians in rating RSI, using the two‐way random effects model and “single rater” unit. The analyses were performed using R Statistical Software (version 4.1.1; R Core Team, 2021) and Stata (version 17; StataCorp).

## 3 RESULTS

In the cancer cohort, 609 patients were hospitalized for COVID‐19 between March 2020 and March 2021, of whom 430 were alive at 3 months after hospital discharge (Figure 1). Of these 430, 98 patients were seen in the MDACC post‐COVID‐19 clinic between June 2020 and June 2021. Of these 98, four patients were excluded because ILAs could not be properly evaluated: two patients lacked HRCT imaging, and two patients had new pneumonia not related to COVID‐19. The final cancer cohort used in our analyses consisted of 94 patients. The initial non‐cancer cohort included 295 patients self‐referred to a post‐COVID‐19 clinic (COE‐UTH) between June 2020 and June 2021. We excluded 182 patients who were not hospitalized and 38 who were hospitalized at another facility and did not have inpatient data available for analyses. Of the remaining 75 patients, 30 were excluded because HRCT was not performed after hospital discharge. The final non‐cancer cohort included 45 patients eligible for analyses. None of the patients had more than one episode of SARS‐CoV‐2 infection.

The characteristics of the study cohort are presented in Table 3. Out of 94 cancer patients, 28 patients (30%) had complete resolution of infiltrates on chest HRCT at 3 months after hospitalization. 20 (21%) of these 28 patients did not report any respiratory symptoms on follow‐up clinic visit (Group A), while 8 had persistent symptoms of cough or shortness of breath (Group B, 9%). 66 (70%) patients had persistent opacities at 3 months after discharge and were considered to have persistent ILAs. Of these, 42 had ILAs at 3 months but not 6 months after hospital discharge (Group C, 44%), while 24 had persistent ILAs at 6 months after hospital discharge (Group D, 26%). Among patients in Group C or D, 39/66 (59%) had persistent respiratory symptoms at 3 months after hospital discharge. In the non‐cancer cohort, after excluding 14 patients who were not evaluable at 6 months post‐discharge, 26/31 (84%) had evidence of ILAs at 3 months (Group C), and 17/26 (65%) had active respiratory symptoms. Of these 31 patients, 14/31 (45%) had evidence of ILAs at 6 months (Group D). Patient‐reported respiratory symptoms included dyspnea (77%), cough (39%) or chest pain/tightness (13%), followed by non‐respiratory symptoms including fatigue (60%), GI disturbances (7%), anosmia (3%), sleep disturbances (2%), or anxiety (2%) (Table S1). Pulmonary function data in patients with ILAs are available in the Online Supplement.

**TABLE 3:** Characteristics of the study cohort.

	Cancer	Non‐cancer
Demographics	*N* = 94	*N* = 45
Age (range), years	60 (50–69)	54 (45–65)
Sex, male, *N* (%)	50 (53)	22(49)
Race, *N* (%)		
White	60 (64)	12 (26)
Black	9 (10)	10 (22)
Hispanic	22 (23)	16 (36)
Asian	3 (3)	3 (7)
Others	0 (0)	4 (7)
Laboratory data, median (IQR)		
White blood count, ×10^9^/L	4.3 (2.4–6.5)	6.0 (4.6–8.6)
Hemoglobin, g/L	11.5 (9.1–13.0)	13.4 (12.0–14.8)
Hematocrit, %	35 (28–39)	41 (36–43)
Platelet count, ×10^9^/L	157 (103–221)	191 (156–248)
Neutrophil count, ×10^9^/L	3.0 (1.4–4.5)	5.0 (3.4–7.1)
Lymphocyte count, ×10^9^/L	0.7 (0.5–1.1)	0.7 (0.9–1.1)
PT, s	14.0 (13.3–14.8)	13.2 (12.6–14.5)
INR	1.11 (1.05–1.22)	1.0 (0.94–1.12)
PTT, s	33 (29–36)	33 (30–37)
Peak fibrinogen, mg/dL	611 (513–683)	635 (562–737)
Peak CRP, mg/dL	103 (48–181)	106 (64–168)
Peak ESR, mm/h	74 (50–98)	65 (39–87)
Peak ferritin, mg/dL	1441 (468–3073)	496 (237–1368)
Peak LDH, U/L	322 (251–428)	443 (301–549)
Peak D‐dimer, μg/mL	1.1 (0.7–2.2)	1.2 (0.7–2.0)
CXR data, median (IQR)		
CXR RSI score on admission	13.0 (6.5–21.6)	19.5 (11.0–29.9)
Peak CXR RSI score	21.0 (15.3–30.8)	26.5 (18–38.8)
Clinical severity data		
WHO clinical progression scale, *N* (%)		
4	17 (18)	4 (9)
5	49 (52)	2 (4)
6	24 (25)	15 (33)
7	1 (1)	0 (0)
8	2 (2)	1 (2)
9	1 (1)	3 (7)
Charlson Comorbidity Index, median (IQR)	19.5 (11–30)	54 (44.5–65)

Abbreviations: CRP, C‐reactive protein; CXR, chest radiograph; ESR, erythrocyte sedimentation rate; ILA, interstitial lung abnormalities; INR, international normalized ratio; IQR, interquartile range; LDH, lactate dehydrogenase; PT, prothrombin time; PTT, partial prothrombin time; RSI, Radiographic Severity Index; WHO, World Health Organization.

### 3.1 Persistent ILAs at after hospital discharge in cancer patients

Univariable predictors of 3‐month ILAs in cancer patients (Groups C and Group D) are reported in Table 4. We dichotomized age (≥ or <60 years), ferritin (< or ≥1000 mg/dL), LDH (< or ≥300 U/L), and d‐dimer (< or ≥1 μg/mL) because these variables did not meet the linearity assumption for our models. In univariable analyses, ferritin ≥1000 (OR 3.0, 95% confidence interval [CI], 1.2–7.5, *p* = 0.02), RSI score on admission (OR 1.5, 95% CI, 1.0–2.1, *p* = 0.04) and peak RSI (OR 1.5, 95% CI, 1.2–2.0, *p* = 0.003) were associated with ILAs at 3 months. Intraclass correlation coefficient was 0.80 for admission RSI (95% CI 0.71–0.86) and 0.82 for peak RSI (95% CI 0.76–0.87), indicating good agreement between readers. Cancer type (solid vs. hematologic) was not associated with the risk for post‐COVID‐19 ILAs. Multivariable logistic regression models showed that age ≥60 years (OR 3.7, 95% CI 1.1–11.8, *p* = 0.03) and peak RSI (OR 1.5 per 5‐point increase, 95% CI 1.1–1.9, *p* = 0.004) associated with ILAs 3 months after hospital discharge. A multivariable model for persistent 6‐month ILAs found that only higher peak RSI (OR 1.3 per 5‐point increase, 95% CI 1.1–1.6; *p* = 0.01) during the hospital course were significantly associated with 6‐month ILA persistence (Table S3). Models including non‐cancer patients found similar results and are reported in Table S4, and the diagnostic performances of models with cancer patients are reported in Table S5.

**TABLE 4:** Univariable and multivariable analysis of cancer cohorts with and without interstitial lung disease at 3 months.

	Univariate analysis crude OR (95% CI)	*p* value	Multivariate analysis adjusted OR (95% CI)	*p* value
Demographics				
Age ≥60 years	1.7 (0.7–4.2)	0.24	3.7 (1.1–11.8)	**0.03**
Sex		0.39		
Male	1.5 (0.6–3.6)			
Female	Reference			
Type of cancer		0.55		
Hematological tumor	0.8 (0.3–2.0)			
Solid tumor	Reference			
Intrathoracic malignancy	7.2 (0.3–158)	0.21		
Prior immunotherapy	2.2 (0.3–19.9)	0.48		
Prior thoracic radiation	4.1 (0.2–110.8)	0.40		
Chemotherapy within 30 days	1.0 (0.4–2.3)	0.93		
Laboratory data				
White blood count	1.0 (0.9–1.1)	0.88		
Hemoglobin	0.8 (0.6–1.0)	0.03		
Hematocrit	0.9 (0.9–1.0)	0.02		
Platelet count	1.0 (0.9–1.0)	0.28		
Neutrophil count	0.9 (0.8–1.1)	0.32		
Lymphocytes	1.1 (0.9–1.3)	0.47		
PT	1.0 (0.8–1.2)	0.84		
PTT	1.0 (1.0–1.1)	0.48		
Peak fibrinogen	1.0 (1.0–1.0)	0.81		
Peak CRP	1.3 (1.0–1.8)	0.06		
Peak ESR	1.1 (0.9–1.2)	0.40		
Peak ferritin ≥1000 mg/dL	3.0 (1.2–7.5)	**0.02**		
Peak LDH ≥300 U/L	2.1 (0.8–5.1)	0.12		
Peak D‐dimer ≥1 μg/mL	2.3 (0.9–5.7)	0.08		
Radiographic data				
CXR RSI score on admission	1.5 (1.0–2.1)	0.04		
Peak CXR RSI score	1.5 (1.2–2.0)	**0.003**	1.5 (1.1–1.9)	**0.004**
Clinical severity data				
WHO clinical progression scale		0.11		
4–5	Reference			
6–9	2.5 (0.8–7.3)			
Charlson Comorbidity Index	1.0 (0.9–1.3)	0.65		

Abbreviations: CRP, C‐reactive protein; CXR, Chest radiograph; ESR, erythrocyte sedimentation rate; ILA, interstitial lung abnormalities; INR, international normalized ratio; LDH, lactate dehydrogenase; OR, odds ratio; PT, prothrombin time; PTT, partial prothrombin time; RSI, Radiographic Severity Index; WHO, World Health Organization. Bold just indicates statistically significant.

### 3.2 Radiologic characteristics of ILAs at 3 and 6 months after hospital discharge

Table 5 compares the prevalence of specific ILAs at 3 and 6 months after hospital discharge only in cancer patients. Air trapping at 3 months was negatively associated persistent ILA at 6 months (OR 0.3, 95% CI 0.1–0.9), but other 3‐month ILAs were not associated with persistent ILAs at 6 months. Fibrotic ILAs, including reticulation, traction bronchiectasis, and architectural distortion, were more common in those with persistent ILAs at 6 months than those with ILAs at 3 months, regardless of 6‐month status (60% at 3 months vs. 88% at 6 months, *p* = 0.01). Honeycombing and cystic changes were rare throughout the cohort observation period.

**TABLE 5:** Pattern of post‐COVID‐19 ILAs at 3 and 6 months after hospital discharge in cancer patients.

	3 months	6 months
Number of ILAs, *N*	65	24
Reticulation, *N* (%)	38 (58)	19 (79)
Consolidation	20 (30)	5 (21)
Ground glass opacity	60 (92)	21 (87)
Traction bronchiectasis	27 (41)	13 (54)
Architectural distortion	12 (18)	7 (29)
Honeycombing	1 (0)	1 (0)
Cysts	4 (6)	3 (4)
Air trapping	26 (40)	9 (37)

One patient found to have ILD at 6 months post‐COVID‐19 lacked a matching 3‐month CT scan.

## 4 DISCUSSION

Our study demonstrates that a substantial proportion of cancer patients who survived to hospital discharge during the initial waves of COVID‐19 had changes consistent with ILAs at 3 months. While most ILAs resolved by 6 months, about 25% of patients had persistent ILAs, and these patients were more likely to have impaired gas diffusion at 3 months after hospital discharge and to have fibrotic ILAs at 6 months after hospital discharge. Severity of SARS‐CoV‐2 pneumonia, as measured by peak RSI within 28 days of hospital admission predicted the presence of ILAs at 3 and 6 months after hospital discharge. Our study suggests that cancer patients frequently develop ILAs after SARS‐CoV‐2 pneumonia, and that those at risk for persistent ILAs can potentially be identified within the first month after SARS‐CoV‐2 pneumonia, setting the stage for targeted longitudinal surveillance programs.

The primary finding of our current study is that cancer patients had a high prevalence of ILAs at 3 and 6 months after hospital discharge for SARS‐CoV‐2 pneumonia. Cancer patients are often excluded from clinical trials and other studies due to their active malignancy, but they are also at high risk for developing complications from non‐cancer health conditions, such as COVID‐19. Our work reinforces that cancer patients may benefit similarly from post‐COVID‐19 surveillance. In fact, the high rate of mortality after hospital discharge in cancer patients with COVID‐19 may argue for even closer monitoring of this high‐risk group.

The high rate of post‐COVID‐19 ILAs confirms observations noted after the 2003 SARS pandemic, when Antonio and associates demonstrated that 62% of the patients with SARS‐CoV pneumonia had radiologic evidence of fibrosis such as parenchymal bands, traction bronchiectasis and other lung abnormalities at a mean of 37 days after hospital discharge.^16^ Risk factors associated with post‐SARS ILAs included older age, ICU admission, elevated LDH, and peak opacification on chest radiographs. Hui and colleagues reported that 30% of the patients who survived SARS‐CoV infection in 2003 had ILAs at 6 months.^17^ Patients with more severe disease (reflected by a higher peak LDH level) and those that required ICU support were more likely to have persistent ILD. The extent of post‐SARS ILAs was associated with impairments in FVC, TLC, and DLCO.

In our cohort, 70% of patients who followed up in the clinic had abnormal interstitial lung findings at 3 months after COVID‐19 pneumonia. While most had resolution of ILAs at 6 months, a substantial minority (26%) did not. Older age and greater peak severity of pneumonia were the main predictors of ILAs at 3 months after discharge, while only greater peak severity of pneumonia was associated with persistent ILAs at 6 months after discharge. Though peak RSI was the best marker of initial severity, we found similar evidence that patients with 3‐ and 6‐month post‐COVID‐19 ILAs were also more likely to have higher levels of the acute phase reactant ferritin, and had a higher (but not statistically significant) odds of having a WHO severity scale of 6 or greater, indicating severe disease, which support our conclusion that the initial severity of SARS‐CoV‐2 pneumonia is the primary determinant of post‐COVID‐19 ILAs. Though ferritin was associated with 3‐month ILAs in univariable analyses, only RSI remained significant in multivariable models, which is likely a reflection of RSI being a more direct measure of the initial lung injury at the time of SARS‐CoV‐2 infection. Our findings suggest that RSI could be incorporated as a screening criterion for entry into a high‐risk cohort for ILA surveillance, but the accuracy and efficacy of such an effort would need to be confirmed prospectively in future studies.

Our findings in cancer patients are consistent with other studies of primarily non‐cancer patients. For example, in a study of 145 patients with COVID‐19, of whom 109 were hospitalized, about two‐thirds had residual ILAs at follow‐up at about 100 days after the initial pneumonia.^18^ In another study of 208 patients hospitalized for COVID‐19, 64% had abnormal chest HRCT findings at 1 year after hospital discharge.^19^ Age ≥ 50 years and severe initial illness, defined as a respiratory rate ≥ 30 breaths/minute, oxygen saturation ≤ 93% at rest on room air, or arterial partial pressure of oxygen/fraction of inspired oxygen ≤300 mmHg, were associated with post‐COVID‐19 ILAs. In both studies, the predominant ILAs were GGOs and reticulations. A recent large study of 3700 individuals with COVID‐19 who were discharged from UK hospitals found that in 255 patients with imaging at a median of 113 days after hospital discharge, nearly 80% had ILAs,^2^ and in 33 patients with subsequent imaging at a median of 161 days after hospital discharge, 28 (85%) continued to demonstrate ILAs. ILAs were more common among males, individuals aged ≥60 years, and in those who required cardiopulmonary support such as invasive ventilation. Finally, in a study of 144 patients discharged from the hospital after SARS‐CoV‐2 pneumonia in China, 39% of patients had evidence of ILAs 2 years after hospital discharge.^20^

On the other hand, we found that the majority of patients with ILAs at 3 months after hospital discharge have resolution by 6 months after hospital discharge. Whether this reflects differences between patients with and without cancer is unclear. For example, it is possible that cancer patients with very severe SARS‐CoV‐2 are more likely to die during the initial hospitalization, and therefore could not contribute to the burden of ILAs months later. Newer imaging modalities, such as photon‐counting CT^21^ or 129XeMRI^22^ may be more sensitive than conventional CT at identifying subtle ILAs. Because ILAs contribute to the mortality in patients with cancer through an increased risk for drug‐induced pneumonitis and likely other mechanisms,^23^ we suggest that the increased rate of ILAs after COVID‐19 in this population may be an important finding.

The mechanisms underlying post‐COVID‐19 ILAs are not well understood. Autopsy studies have shown that severe SARS‐CoV‐2 pneumonia results in dense alveolar fibrosis^24^ associated with increases in the expression of pro‐fibrotic factors such as transforming growth factor beta (TGFβ) and connective tissue growth factor (CTGF) in alveolar epithelial cells.^25^ MUC5B polymorphisms are well known to be associated with the risk of idiopathic pulmonary fibrosis,^26^ and in a postmortem study of 21 patients who died after COVID‐19, both MUC5B and MUC5AC transcript levels were elevated in the subacute and chronic disease phases, and 93% of COVID‐19 lungs suitable for distal lung studies exhibited MUC5B‐driven mucus accumulation in distal airways.^27^ Interestingly, dexamethasone, which is commonly given to treat SARS‐CoV‐2 pneumonia,^28^ diminished MUC5B‐induced mucus accumulation in an in vitro model, but since most patients already receive dexamethasone to treat COVID‐19, it is unclear whether administering additional upfront broad anti‐inflammatory therapies would necessarily further lower the risk for post‐COVID‐19 fibrosis. Additionally, familial pulmonary fibrosis is associated with mutations that predispose to shorter telomere lengths, such as in the telomere‐related genes TERT and TERC,^29^, ^30^, ^31^ and recent work demonstrates that shorter blood leukocyte telomere length is associated with radiographic ILAs at 4 months after COVID‐19 infection.^5^ We found that while GGOs were the predominant ILA at 3 months after hospital discharge, those with persistent ILAs at 6 months tended to have features of fibrosis, including reticulation and traction bronchiectasis, which is consistent work by Han et al showing that more than half of patients who were seen 2 years after hospital discharge for SARS‐CoV‐2 pneumonia had fibrotic ILAs.^20^ These observations suggest that there may be shared mechanisms whereby SARS‐CoV‐2 may predispose toward persistent ILAs. Future studies are needed to determine whether antifibrotic therapies currently in use to treat interstitial lung diseases also have a role to prevent or treat post‐COVID‐19 fibrosis.

There are several limitations to our analysis. First, while patients were systematically referred to PASC clinics after hospital discharge, the cancer cohort included only 25% of discharged patients, thus possibly resulting in either a survival bias, where healthy survivors were more likely to present to a clinic, or a bias toward more symptomatic patients, both of which may limit generalization of our findings. This former bias was likely to be present in the non‐cancer cohort, where patients self‐referred, typically due to ongoing pulmonary symptoms, and therefore our primary analyses focused on cancer patients. While we performed secondary analyses including non‐cancer patients, these must be interpreted with caution due to the suspected selection bias with self‐referral. In addition, the limited timeframe and the lower rate of post‐discharge follow‐up limited our sample size for analyses. Secondly, about 40% of non‐cancer patients did not have HRCT during the first visit in the PASC clinic and were excluded from this analysis. Third, while we surmise that the higher prevalence of GGOs at 3 months post‐discharge represents transient inflammation in most patients, while the greater prevalence of fibrotic ILAs in patients with persistent ILAs 6 months after discharge represents true fibrosis, we were not able to directly investigate the lung pathology in these cases. Furthermore, we did not perform genomic analyses for mutations related to common interstitial lung diseases or evidence for ongoing inflammation in the blood to confirm whether mechanisms in sporadic interstitial lung diseases and post‐COVID‐19 ILAs were similar. Fourth, we determined the presence of symptoms based upon a clinical evaluation, but did not use a patient‐reported outcome instrument. Fifth, this work was performed during the early period of the pandemic, but as new variants, therapies and vaccinations continue to change the landscape of COVID‐19, further studies may be needed to confirm the incidence of post‐COVID‐19 ILAs. In fact, recent variants (e.g., Omicron subtypes) have shown milder initial radiographic severity.^32^, ^33^ Nevertheless, our study is unique and provides valuable information derived from a multidisciplinary PASC clinic created in the earliest days of the pandemic.

In conclusion, post‐COVID‐19 ILAs are common in cancer patients 3 months after hospital discharge. Though most ILAs resolve by 6 months after hospital discharge, up to a quarter of patients have persistent ILAs, which tend to be more fibrotic. Peak RSI during the initial SARS‐CoV‐2 pneumonia and older age are strong predictors of persistent post‐COVID‐19 ILAs and may allow for more focused post‐COVID‐19 monitoring. As the pandemic continues to evolve, further studies are needed to continually investigate the changing incidence of post‐COVID‐19 ILAs with new variants. Furthermore, there is a need to develop evidence‐based algorithms to diagnose and treat post‐COVID‐19 ILAs, particularly to standardize measurements of RSI and to clarify the role of antifibrotic therapies. Additionally, mechanistic studies that clarify the role of host responses and pathogen‐mediated injury to the lung may illuminate new therapeutic avenues to mitigate post‐COVID‐19 ILAs. Finally, our work shows the need to develop new paradigms for post‐infectious pulmonary surveillance in order to mitigate long‐term morbidity after serious pulmonary infections.

## AUTHOR CONTRIBUTIONS

**Sungryong Noh:** Conceptualization (supporting); data curation (equal); formal analysis (supporting); investigation (equal); validation (equal); writing – original draft (equal); writing – review and editing (equal). **Christopher Bertini:** Conceptualization (equal); data curation (equal); investigation (equal); methodology (equal); validation (equal); writing – original draft (equal); writing – review and editing (equal). **Isabel Mira‐Avendano:** Conceptualization (supporting); data curation (equal); investigation (supporting); project administration (supporting); resources (equal); validation (equal); writing – original draft (supporting); writing – review and editing (supporting). **Maryam Kaous:** Conceptualization (supporting); data curation (equal); investigation (supporting); methodology (supporting); project administration (supporting); resources (equal); supervision (supporting); validation (supporting); writing – original draft (supporting); writing – review and editing (supporting). **Bela Patel:** Conceptualization (supporting); data curation (supporting); investigation (supporting); methodology (supporting); project administration (equal); resources (equal); validation (supporting); writing – original draft (supporting); writing – review and editing (supporting). **Saadia A Faiz:** Conceptualization (supporting); formal analysis (supporting); resources (supporting); validation (equal); writing – original draft (supporting); writing – review and editing (supporting). **Vickie R Shannon:** Resources (supporting); writing – original draft (supporting); writing – review and editing (supporting). **Diwakar Balachandran:** Resources (supporting); writing – original draft (supporting); writing – review and editing (supporting). **Lara Bashoura:** Conceptualization (equal); project administration (supporting); resources (equal); writing – original draft (supporting); writing – review and editing (supporting). **Roberto Adachi:** Writing – original draft (supporting); writing – review and editing (supporting). **Scott Evans:** Resources (supporting); writing – original draft (supporting); writing – review and editing (supporting). **Burton Dickey:** Resources (supporting); writing – original draft (supporting); writing – review and editing (supporting). **Carol C. Wu:** Visualization (equal); writing – original draft (supporting); writing – review and editing (supporting). **Girish S. Shroff:** Visualization (equal); writing – original draft (supporting); writing – review and editing (supporting). **Joanna Grace Manzano:** Conceptualization (equal); data curation (supporting); methodology (supporting); project administration (equal); resources (lead); supervision (equal); writing – original draft (equal); writing – review and editing (equal). **Bruno P Granwehr:** Resources (supporting); writing – original draft (supporting); writing – review and editing (supporting). **Shannon Holloway:** Resources (supporting); writing – original draft (supporting); writing – review and editing (supporting). **Kodwo Dickson:** Resources (equal); writing – original draft (supporting); writing – review and editing (supporting). **Alyssa Mohammed:** Resources (equal); writing – original draft (supporting); writing – review and editing (supporting). **Mayoora Muthu:** Methodology (equal); resources (equal); writing – original draft (supporting); writing – review and editing (supporting). **Hui Song:** Data curation (lead); investigation (supporting); methodology (supporting); resources (lead); writing – original draft (supporting); writing – review and editing (supporting). **D3CODE Team:** Data curation (lead); formal analysis (supporting); methodology (lead); resources (lead); writing – original draft (supporting); writing – review and editing (supporting). **Caroline Chung:** Resources (equal); writing – original draft (supporting); writing – review and editing (supporting). **Jia Wu:** Investigation (supporting); writing – original draft (supporting); writing – review and editing (supporting). **Lyndon Lee:** Data curation (equal); investigation (supporting); methodology (supporting); writing – original draft (supporting); writing – review and editing (supporting). **Ying Jiang:** Data curation (supporting); formal analysis (lead); investigation (equal); methodology (supporting); validation (lead); writing – original draft (supporting); writing – review and editing (supporting). **Fareed Khawaja:** Conceptualization (equal); formal analysis (equal); investigation (equal); project administration (equal); supervision (equal); validation (equal); writing – original draft (equal); writing – review and editing (equal). **Ajay Sheshadri:** Conceptualization (lead); data curation (lead); formal analysis (equal); investigation (lead); methodology (lead); project administration (lead); resources (lead); supervision (lead); validation (lead); writing – original draft (lead); writing – review and editing (lead).

## FUNDING INFORMATION

This research is supported in part by the National Institutes of Health through MD Anderson's Cancer Center Support Grant (CA016672).

**D3CODE Team**: Ashley Aroe, Thomas A. Aloia, Lee Andrews II, Kiran K. Badami, Janna A. Baganz, Pratibha Bajwa, Lora R. Baker, Gregory R. Barbosa, Hannah C. Beird, Matt Bourgeois, Kristy Brock, Elizabeth M. Burton, Juan Cata, Caroline Chung, Michael Cutherell, John Cuenca, Pierre B. Cyr, Bouthaina Dabaja, Hiba Dagher, Kevin M Daniels, Mary Domask, Giulio Draetta, Paul Edelkamp, Jr., Sarah Fisher, Katy Elizabeth French, Andrew Futreal, Maria Gaeta, Christopher Gibbons, Myrna Godoy, Drew Goldstein, Jillian Gunther, Cristhiam Hernandez, Kate Hutcheson, David Jaffray, Jeff Jin, Teny Matthew John, Trey Kell, Mark Knafl, Anai Kothari, Rayson C. Kwan, J. Jack Lee, Yue Liao, Jennifer Litton, Alex Liu, Kevin W. McEnery, Mary McGuire, Benjamin Mescher, Tejo Musunuru, Mayoora Muthu, Joseph Nates, Craig S. Owen, Priyadharshini Padmakumar. Melody Page, Nicholas Palaskas, Jay J. Patel, Sabitha Prabhakaran, Lucas Ramsey, Vinod Ravi, Ludivine Russell, Bilja Sajith, Paul A. Scheet, Stephanie Schmidt, Kenna R. Shaw, Sanjay Shete, Daniel P. Shoenthal, Lessley J. Stoltenberg, Ishwaria Subbiah, Chuck Suitor, Hussein Tawbi, Phillip Thompson, Anastasia Turin, Samir Unni, Benju Vicknamparampil, Max C. Weber, John Weinstein, Zoe Williams, Scott Eric Woodman, Mark C. Wozny, Carol Wu, Jia Wu, James C. Yao, Chingyi Young, Emily Yu, Steven Zatorski.

## CONFLICT OF INTEREST STATEMENT

None.

## Supporting information


**Data S1.** Supporting Information

## Data Availability

De‐identified data will be provided upon request for investigators with appropriate study plans.
