# Supplementary material for: Interstitial lung abnormalities after hospitalization for COVID‐19 in patients with cancer: A prospective cohort study
Source: Cancer Med. 2023 Aug 18;12(17):17753–65. doi: 10.1002/cam4.6396 (PMC10524033; doi:10.1002/cam4.6396)
Supplement: Supplementary file 1 — Data S1. Supporting Information [file CAM4-12-17753-s001.docx]

**Interstitial lung abnormalities after COVID-19 in patients with and without cancer: a prospective cohort study (supplement)**

**Results**

*Symptom Burden*

Patient-reported respiratory symptoms among those with ILAs at 3 months included dyspnea (77%), cough (39%) or chest pain/tightness (13%), followed by non-respiratory symptoms including fatigue (60%), GI disturbances (7%), anosmia (3%), sleep disturbances (2%), or anxiety (2%) (**Supplementary Table 1)**.

*Association of ILAs with pulmonary function testing*

Patients with persistent ILAs at 6 months had modestly lower DLCO at 3 months after hospital discharge as compared to patients without persistent ILAs at 6 months (**Supplementary Table 2**), but spirometry and lung volumes were otherwise not significantly different. Similarly, six-minute walk test distance and oxygen saturation nadir (SpO2) at the 3-month visit did not differ between patients with and without 6-month ILAs. Peak RSI and 3-month DLCO were modestly correlated in cancer patients (Spearman’s coefficient: -0.45, p<0.001).

*Persistent ILAs at 6 months after hospital discharge in cancer patients.*

Univariable predictors of 6-month ILAs in cancer patients (Groups D) are reported in **Supplementary Table 3.** We again dichotomized age (≥ or <60 years), ferritin (< or ≥1000 mg/dL), LDH (< or ≥300 units/L), and d-dimer (< or ≥1 ug/mL) because these variables did not meet the linearity assumption for our models. In univariable analyses, LDH ≥300 units/L (OR 3.1, 95% CI 1.1-8.7) and higher peak RSI (OR 1.3 per 5-point increase, 95% CI 1.1-1.6) were associated with ILAs at 6 months, but a multivariable logistic regression model for persistent 6-month ILAs found that only higher peak RSI (OR 1.3 per 5-point increase, 95% CI 1.1 – 1.6; p= 0.01) during the hospital course were significantly associated with 6-month ILA persistence.

*Persistent ILAs at 3 months after hospital discharge in the combined cohort of cancer and non-cancer patients*

Univariable predictors of 3-month ILAs in cancer and non-cancer patients (Groups C and Group D) are reported in **Supplementary Table 4.** We dichotomized age (≥ or <60 years), ferritin (< or ≥1000 mg/dL), LDH (< or ≥300 units/L), and d-dimer (< or ≥1 ug/mL) because these variables did not meet the linearity assumption for our models. In univariable analyses, higher peak values of CRP (OR 1.5, 95% CI, 1.1-2.0, p= 0.01), LDH (OR 3.0, 95% CI, 1.3-6.9, p=0.01) and D-dimer (OR 2.3, 95% CI, 1.0-5.3, p=0.04) were associated with development of ILAs at 3 months after hospital discharge. Additionally, RSI score on admission (OR 1.4 per 5-point increase, 95% CI, 1.1-2.0, p=0.02), peak RSI (OR 1.5, 95% CI, 1.2-1.9, p<0.001) and WHO clinical progression scale (OR 2.8 per 1-point increase, 95% CI, 1.1-7.5, p=0.04) also correlated with the presence of ILAs 3 months following hospital discharge. After variable selection, the multivariable model showed only two factors associated with development of ILAs at 3 months after hospital discharge: age ≥60 (OR 4.4, 95% CI, 1.5-12.7, p= 0.006) and peak RSI (OR 1.5 per 5-unit increase, 95% CI 1.2-1.9, p= 0.0005).

*Diagnostic performance of RSI thresholds*

**Supplementary Table 5** shows areas under the receiver-operator curve (AUC) for optimal thresholds of peak RSI within 28 days of initial SARS-CoV-2 infection to predict 3- and 6-month ILA persistence using the models featuring only cancer patients. The optimal cutoff value for peak admission RSI was 18, with an AUC of 0.75 (95% CI 0.64-0.85) for all patients, but higher AUC in patients aged <60 years (0.80, 95% CI 0.68-0.92) as compared to patients aged greater than 60 years (AUC 0.63, 95% CI 0.59-0.96). The optimal cutoff for the prediction of ILAs at 6 months was between 21-29, but the AUC for the prediction of 6-month ILAs was lower (optimal threshold 25, AUC 0.59, 95% CI 0.42-0.74). The performance of peak RSI for the prediction of 6-month ILAs was greater in patients younger than 60 years (optimal threshold 29, AUC 0.77, 95% CI 0.59-0.96) as compared to patients aged greater than 60 years (optimal threshold 21, AUC 0.58, 95% CI 0.42-0.74). For patients aged <60 years, the sensitivity and specificity for ILAs with an RSI greater than the optimal thresholds were 79% and 70% for 3-month ILAs and 82% and 67% for 6-month ILAs.

**Supplementary Table 1. Symptom profile in all patients with ILAs and respiratory symptoms at 3 and 6 months after hospital discharge**

|  | **3-month ILAs** | **6-month ILAs** |
| --- | --- | --- |
| **Number of patients who had residual symptoms *- N*** | 48 | 26 |
| **Symptoms *– N (%)*** |  |  |
| **Cough** | 18 (38) | 9 (35) |
| **Dyspnea** | 38 (79) | 20 (77) |
| **Fatigue** | 30 (63) | 13 (50) |
| **Chest pain/tightness** | 7 (13) | 3 (12) |
| **GI Disturbances** | 4 (15) | 1 (4) |
| **Anosmia** | 2 (4) | 0 (0) |
| **Sleep Disturbances** | 0 (0) | 2 (8) |
| **Anxiety/Depression** | 1 (2) | 0 (0) |

**Supplementary Table 2. Pulmonary Function Test and Six-Minute Walk Test Data between Cohorts with and without Interstitial Lung Abnormalities at 6 months after hospital discharge**

|  | ILAs  (n=38) | No ILAs  (n=101) | p - value |
| --- | --- | --- | --- |
| PFT, median (IQR) |  |  |  |
| FVC % predicted | 80 (66-95) | 85 (68-95) | 0.74 |
| DLCO % predicted | 63 (53-77) | 74 (62-86) | 0.04 |
| TLC % predicted | 82 (70-93) | 88 (76-100) | 0.24 |
| 6MWT, median (IQR) |  |  |  |
| Distance (meter) | 373 (323-457) | 365 (277-430) | 0.33 |
| SpO₂ nadir% | 95 | 97 (93-98) | 0.54 |

PFT, pulmonary function test; IQR, interquartile range; FVC, forced vital capacity; DLCO, diffusion capacity carbon monoxide; TLC, total lung capacity; 6MWT, Six-Minute Walk Test; SpO_2_, saturation of peripheral oxygen

**Supplementary Table 3. Univariable and Multivariable Predictors of ILAs in Cancer Patients at 6 Months**

|  | Univariable analysis  Crude OR (95% CI) | *p* - value | Multivariable analysis  Adjusted OR (95% CI) | *p* - value |
| --- | --- | --- | --- | --- |
| Demographics | | | | |
| Age ≥ 60 years | 1.4 (0.6-3.6) | 0.48 |  |  |
| Sex  Male  Female | \| 1.7 (0.7-4.3) \| \| --- \| \| Reference \| | 0.29 |  |  |
| Type of Cancer  Hematologic  Solid tumor | \| 1.3 (0.5-3.5) \| \| --- \| \| Reference \| | 0.65 |  |  |
| Intrathoracic Malignancy | 0.5 (0.1-4.1) | 0.49 |  |  |
| Prior immunotherapy | 1.5 (0.3-8.8) | 0.65 |  |  |
| Prior thoracic radiation | 0.3 (0.01-8.2) | 0.48 |  |  |
| Chemotherapy within 30 days | 0.8 (0.3-2.1) | 0.64 |  |  |
| Laboratory Data | | | | |
| White blood count | 1.1 (1.0-1.2) | 0.15 |  |  |
| Hemoglobin | 0.8 (0.7-1.0) | 0.07 |  |  |
| Hematocrit | 0.9 (0.9-1.0) | 0.07 |  |  |
| Platelet count | 1.0 (0.9-1.0) | 0.28 |  |  |
| Neutrophil count | 0.8 (0.7-1.0) | 0.13 |  |  |
| Lymphocytes | 1.3 (0.8-2.1) | 0.25 |  |  |
| PT | 0.9 (0.7-1.1) | 0.16 |  |  |
| PTT | 1.0 (1.0-1.1) | 0.56 |  |  |
| Peak Fibrinogen | 1.0 (1.0-1.0) | 0.79 |  |  |
| Peak CRP | 1.2 (0.9-1.6) | 0.18 |  |  |
| Peak ESR | 1.1 (1.0-1.3) | 0.12 |  |  |
| Peak Ferritin ≥ 1,000 mg/dL | 3.1 (1.1-8.7) | 0.03 |  |  |
| Peak LDH ≥ 300 U/L | 1.4 (0.5-3.5) | 0.53 |  |  |
| Peak D-dimer ≥ 1 ug/mL | 1.4 (0.5-3.9) | 0.46 |  |  |
| Radiographic Data | | | | |
| CXR RSI score on admission | 0.9 (0.7-1.2) | 0.46 |  |  |
| Peak CXR RSI score | 1.3 (1.0-1.6) | 0.02 | 1.3 (1.1-1.6) | 0.014 |
| Clinical Severity Data |  |  |  |  |
| WHO Clinical Progression Scale  4-7  8-9 | \| Reference \| \| --- \| \| 1.5 (0.1-17) \| | 0.75 |  |  |
| FVC% at 3 months | 1.0 (1.0-1.0) | 0.44 |  |  |
| DLCO% at 3 months | 1.0 (0.9-1.0) | 0.09 |  |  |
| TLC% at 3 months | 1.0 (1.0-1.0) | 0.17 |  |  |
| 6MWT distance at 3 months (per m) | 1.0 (1.0-1.1) | 0.22 |  |  |
| Sp0_2_ nadir% at 3 months (per 1% increase) | 0.9 (0.8-1.1) | 0.23 |  |  |

*ILA, interstitial lung abnormalities; OR, odds ratio; PT, prothrombin time; INR, international normalized ratio; PTT, partial prothrombin time; CRP, C-reactive protein; ESR, erythrocyte sedimentation rate; LDH, lactate dehydrogenase; CXR, Chest radiograph; RSI, radiographic severity index; WHO, World Health Organization; FVC, forced vital capacity; DLCO, diffusing capacity for carbon monoxide; TLC, total lung capacity; 6MWT, 6 minute walk test; Sp0_2_, saturation of peripheral oxygen

**Supplementary Table 4. Univariable and Multivariable Predictors of ILAs in Cancer and non-cancer Patients at 3 Months**

|  | Univariable analysis  Crude OR (95% CI) | *p* - value | Multivariable analysis  Adjusted OR (95% CI) | *p* - value |
| --- | --- | --- | --- | --- |
| Demographics | | | | |
| Age ≥ 60 years | 1.7 (0.7-4.2) | 0.24 | 3.7 (1.1-11.8) | **0.03** |
| Sex  Male  Female | 1.5 (0.6-3.6)  Reference | 0.39 |  |  |
| Laboratory Data | | | | |
| White blood count | 1.0 (0.9-1.1) | 0.88 |  |  |
| Hemoglobin | 0.8 (0.6-1.0) | 0.03 |  |  |
| Hematocrit | 0.9 (0.9-1.0) | 0.02 |  |  |
| Platelet count | 1.0 (0.9-1.0) | 0.28 |  |  |
| Neutrophil count | 0.9 (0.8-1.1) | 0.32 |  |  |
| Lymphocytes | 1.1 (0.9-1.3) | 0.47 |  |  |
| PT | 1.0 (0.8-1.2) | 0.84 |  |  |
| PTT | 1.0 (1.0-1.1) | 0.48 |  |  |
| Peak Fibrinogen | 1.0 (1.0-1.0) | 0.81 |  |  |
| Peak CRP | 1.3 (1.0-1.8) | 0.06 |  |  |
| Peak ESR | 1.1 (0.9-1.2) | 0.40 |  |  |
| Peak Ferritin ≥ 1,000 mg/dL | 3.0 (1.2-7.5) | **0.02** |  |  |
| Peak LDH ≥ 300 U/L | 2.1 (0.8-5.1) | 0.12 |  |  |
| Peak D-dimer ≥ 1 ug/mL | 2.3 (0.9-5.7) | 0.08 |  |  |
| Radiographic Data | | | | |
| CXR RSI score on admission | 1.5 (1.0-2.1) | 0.04 |  |  |
| Peak CXR RSI score | 1.5 (1.2-2.0) | **0.003** | 1.5 (1.1-1.9) | **0.004** |
| Clinical Severity | | | | |
| WHO Clinical Progression Scale  4-5  6-9 | Reference  2.5 (0.8-7.3) | 0.11 |  |  |
| Charlson Comorbidity Index | 1.0 (0.9-1.3) | 0.65 |  |  |

*ILA, interstitial lung abnormalities; OR, odds ratio; PT, prothrombin time; PTT, partial prothrombin time; CRP, C-reactive protein; ESR, erythrocyte sedimentation rate; LDH, lactate dehydrogenase; CXR, Chest radiograph; RSI, radiographic severity index; WHO, World Health Organization

**Supplementary Table 5. Sensitivity and Specificity of Radiographic Severity Index Cutoffs for the Prediction of 3- and 6-month ILAs in Cancer Patients**

|  | Area under the curve | Optimal cut-off value | Sensitivity (95% CI) | Specificity (95% CI) |
| --- | --- | --- | --- | --- |
| All Ages  ILAs at 3 months  ILAs at 6 months | 0.75 (0.64-0.85)  0.65 (0.53-0.77) | 18  25 | 0.72 (0.62-0.80)  0.59 (0.42-0.74) | 0.65 (0.46-0.81)  0.62 (0.51-0.71) |
| Patients ≥60 years  ILAs at 3 months  ILAs at 6 months | 0.63 (0.40-0.85)  0.58 (0.42-0.74) | 19  21 | 0.55 (0.41-0.68)  0.57 (0.37-0.76) | 0.67 (0.30-0.90)  0.65 (0.48-0.79) |
| Patients <60 years  ILAs at 3 months  ILAs at 6 months | 0.80 (0.68-0.92)  0.77 (0.59-0.96) | 18  29 | 0.79 (0.65-0.89)  0.82 (0.52-0.95) | 0.70 (0.48-0.85)  0.67 (0.54-0.78) |

*ILAs, interstitial lung abnormalities; CI, confidence interval*
